# Intestinal immunity marches on its stomach

**DOI:** 10.1186/1479-5876-10-S3-I9

**Published:** 2012-11-28

**Authors:** Marc Veldhoen

**Affiliations:** 1 Babraham Institute, Lymphocyte Dignalling and Development Laboratory-Babraham Research Campus, Cambridge, UK

## 

In addition to genetic makeup, age, and gender, our diet has a major impact on the immune status and the intestinal microbiota. In return, alterations in the latter and their metabolites affect both local and systemic immunity. The microbiota and diet contribute important substrates, enabling the generation of essential compounds like vitamins that support the biosynthesis of many products throughout the body. As such, the diet constitutes a major contributor to the composition and maintenance of cells of the immune system and health status of the host. Immune protection against invading microorganisms and their products is often initiated at epithelial barrier sites such as the intestine. At these sites reside specialised lymphocytes that are important not only as a first line of defence but also in epithelial barrier organisation and wound repair.

The transcription factor aryl hydrocarbon receptor (AhR) is selectively expressed in some immune cells, importantly in Th17, gamma/delta T cells and innate lymphoid cells. All these cell types are found, and often enriched, at barrier sites. The AhR contains a paired Per-Arnt-Sim (PAS) domain, highly conserved in proteins involved in sensing environmental change. Its activation is important for optimal development of the Th17 cell subset and results in enhanced activation of both Th17 and IL-17 producing gamma/delta T cells and their capacity to produce IL-22. Especially at epithelial sites, AhR activation in response to endogenous and exogenous ligands may constitute a way in which environmental stimuli could affect the immune status. Surprisingly, we found that elements from the diet are responsible for the activation of AhR in intestinal immune cells.

